# Physicial Benefit of Sunday

**Published:** 1877-05

**Authors:** 


### THE PHYSICAL BENEFIT OF SUNDAY.

Sunday is God's special present to the working-man, and one of His chief objects is to prolong his life, and preserve efficient his working tone. In the vital system it works like a compensation pound; it replenishes the spirit, the elasticity and vigor, which the last six days have drained away, and supplies the force which is to fill the six days succeeding; and in the economy of existence it answers the same purpose as the economy of income is answered by a savings bank. The frugal man who puts away a pound to-day, and another pound next month, and who, in a quiet way, is putting by his stated pound from time to time, when he grows old and frail gets not only the same pound back again, but a good many pounds besides. And the conscientious man, who husbands one day of his existence every week—who, instead of allowing Sunday to be trampled and torn in the hurry and scramble of life, treasures it up—the Lord of Sunday keeps it for him, and in length of days the hail of age gives it back with usury. The savings bank of human existence is the weekly Sunday.

